# Health Promotion in Glycemic Control and Emotional Well-Being of People with Type 1 Diabetes Mellitus: A Systematic Review and Meta-Analysis

**DOI:** 10.3390/healthcare12232461

**Published:** 2024-12-06

**Authors:** Miguel Garrido-Bueno, Manuel Pabón-Carrasco, Nerea Jiménez-Picón, Rocío Romero-Castillo

**Affiliations:** 1 Red Cross Nursing University Centre, University of Seville (Seville), 41009 Sevilla, Spain; 2 Department of Nursing, Faculty of Nursing, Physiotherapy and Podiatry, University of Seville (Seville), 41009 Sevilla, Spain; mpabon2@us.es (M.P.-C.); njimenez@us.es (N.J.-P.)

**Keywords:** type 1 diabetes mellitus, patient education, health promotion, glycemic control, emotional well-being

## Abstract

Background/Objectives: Structured therapeutic patient education is the key to improving biopsychosocial outcomes in people with type 1 diabetes mellitus. This study aimed to determine the effects of structured therapeutic education on glycemic control and emotional well-being in people with type 1 diabetes mellitus. Methods: This is a systematic review with a meta-analysis (PROSPERO ID: CRD42023390079). Searches were performed in Scopus, MEDLINE, Web of Science, CINAHL, APA PsycInfo, APA PsycArticles, and the Psychology Database (June–August 2024). The eligibility criteria included randomized controlled trials published in English or Spanish within the past 10 years. Data extraction and risk of bias evaluations were independently conducted by two reviewers. The outcomes analyzed included glycated hemoglobin, time in range, emotional well-being, self-management behaviors, and adherence to treatment. Meta-analyses were performed using RevMan with random and fixed effects models. Results: Seventeen studies met the eligibility criteria. There was a significant improvement in glycemic control, stress, anxiety, and treatment satisfaction, although the results for the other emotional outcomes were mixed. Conclusions: Structured therapeutic patient education improves glycemic control and selected emotional outcomes in individuals with type 1 diabetes mellitus. Further trials are needed to refine the characteristics of the intervention and broaden the applicability of the findings to diverse populations.

## 1. Introduction

Type 1 diabetes mellitus (T1DM) is a public health problem concern as it has a very high prevalence and economic cost worldwide [1]. It is caused by the loss of pancreatic beta cells, which results in insulin deficiency [2]. People with diabetes can have symptoms such as polydipsia, polyuria, and polyphagia [3], and a marked psychosocial impact consisting of stress, depression, anxiety, eating disorders, family issues, and a loss of quality of life [4,5].

This disease has an ever-growing nursing interest due to its prognosis, as people who have it are at greater risk of suffering from both acute and chronic complications than people who do not. Some of these complications are hyperglycemia, hypoglycemia, ketoacidosis, diabetic foot, member amputations, neuropathies, retinopathies, nephropathies, and cardiovascular diseases, which could even lead to death [5].

Nurse-led therapeutic patient education (TPE) is key to the holistic follow-up of people with T1DM [6]. It is defined as some ‘educational activities that are essential for the management of pathological conditions, designed to help people and their families manage their treatment and prevent avoidable complications’ [7]. In T1DM, nurses should focus on glycemic control, a social support system, health determinants [4], adherence to treatment, quality of life, well-being, and self-efficacy of their patients [6]. In fact, education and support are key to the care of this disease [8].

NANDA International (NANDA-I), the Nursing Outcomes Classification (NOC), and the Nursing Intervention Classification (NIC) taxonomies show that T1DM-related TPE is integrated in every step of the nursing process [9,10,11]. Concretely, NIC presents interventions such as Teaching: prescribed diet (5614), Teaching: prescribed exercise (5612), or Help with self-care (1800) [11]. Furthermore, Dorothea Orem’s self-care deficit nursing theory also mentions TPE, as it defines self-care as an ‘activity learned by people, which is oriented toward themselves, others, or the environment, to regulate the factors that affect their development in their health, life, or well-being’ [12].

In the current literature, different systematic reviews and meta-analyses have shown the possible effect of digital interventions on diabetes [13], and the impact of education programs on glycemic control for people and their families [14]. In these cases, neither of the two studies differentiate their results in terms of the type of diabetes that the participants in their sample had [13,14].

A recent Cochrane meta-analysis establishes that a significant improvement was found in the Hb A1c and body weight of people with diabetes and obesity after a TPE intervention, but could not reach statistical significance in many psychosocial outcomes [15]. This is confirmed by another Cochrane meta-analysis that showed statistical significance in the pooled effect of TPE but did not indicate it in body weight and quality of life or behavioral-related outcomes [16].

Regarding a meta-analysis by the Agency for Healthcare Research and Quality (AHRQ), a reduction in Hb A1c and an active glycemic control were shown to be statistically significant 6 months after the intervention in people who underwent a TPE program, but it should be noted that some included studies had small sample sizes and that the review was published more than 5 years ago [17]. A similar issue was found in a systematic review by the National Institute for Health and Care Excellence (NICE), in which, although some TPE trials were effective in improving glycemic control in people with glycemic problems, its results included studies dating back more than 30 years [18].

Although the influence of TPE on people with diabetes has previously been shown, a new meta-analysis is needed to assess the effects of the intervention specifically on people with T1DM [6], instead of including a sample with different types of diabetes and concomitant diseases in the same review, without any clear differentiation in their results or discussion [13,14,15]. Due to the considerable progress that has taken place in the field of study since previous reviews [19], it is essential to develop a comprehensive new systematic review and meta-analysis rather than simply updating the old ones. This will ensure that the latest advancements and changes are fully addressed.

Due to all of this, a Population, Intervention, Comparison, and Outcomes (PICO) research question was established: ‘Does TPE improve glycemic control and emotional well-being in people with T1DM compared with standard care?’ Subsequently, the aim of this study was to determine the effects of structured TPE on glycemic control and emotional well-being in people with T1DM.

## 2. Materials and Methods

### 2.1. Design

This study is a systematic review with a meta-analysis. An alternative hypothesis (H1) was established, stating that TPE improves glycemic control and certain psychosocial outcomes in people with T1DM. A study protocol was registered in the International Prospective Register of Systematic Reviews (PROSPERO) (ID: CRD42023390079) [20], which was modified to include the meta-analysis, as it was not initially planned to be carried out.

### 2.2. Search Methods

The expressions used in a PICO research question [21] were adapted to terms from the Medical Subject Headings (MeSH) thesaurus (National Library of Medicine, 2023), modified using truncations (*), and linked using Boolean operators (AND, OR, NOT) to generate a search strategy that would be used in different databases such as Scopus, PubMed (MEDLINE), Web of Science, CINAHL, APA PsycInfo, APA PsycArticles, and the Psychology Database (Table 1). Searches in all these databases were performed from June to August 2024. No studies were included by additional means, and the search was not updated after downloading the files from the databases.

### 2.3. Eligibility

The inclusion and exclusion criteria were established according to the elements of the PICO research question (Table 2). Only randomized controlled trials (RCTs) were included to achieve a high level of evidence, which was evaluated using the Scottish Intercollegiate Guidelines Network (SIGN) scale [22]. Systematic reviews were not included as this was not a systematic review of reviews, so primary studies were screened instead for inclusion. Pregnant patients were excluded to prevent bias. All the trials selected for analysis were available in English.

### 2.4. Screening Process

The selection of RCTs was independently performed by two reviewers, from June to August 2024. They (a) designed and implemented a standardized search strategy across multiple databases; (b) independently reviewed titles, abstracts, and full texts, resolving discrepancies through consensus; and (c) extracted data and assessed the quality of the selected studies using a validated tool. This reference selection process included duplicate removal using Zotero version 6.0.36., was backed up in Microsoft Excel 2016, and was represented in a Preferred Reporting Items for Systematic reviews and Meta-Analyses (PRISMA) 2020 flow diagram [23].

### 2.5. Quality Appraisal

The review was carried out following the Cochrane Handbook for Systematic Reviews of Interventions methods manual [24]. The quality of the research was independently measured by two reviewers using the PRISMA 2020 Checklist (Supplementary S1), the PRISMA 2020 for Abstracts Checklist, the PRISMA-S Checklist [23], and the Assessing the Methodological Quality of Systematic Reviews (AMSTAR) 2 [25] reporting guidelines. Any possible discrepancies between the reviewers were resolved by consensus. The Synthesis Without Meta-analysis (SWiM) reporting guideline was not used as this systematic review featured useful data for conducting a meta-analysis [26].

The risk of bias of the included studies was independently evaluated by two reviewers using the Cochrane risk of bias 2 tool (RoB 2) because this was a review of intervention trials [27]. This tool assesses the methodological quality of studies by evaluating biases in specific domains, including selection, performance, detection, attrition, reporting, and others, classifying them as “low risk”, “some concerns”, or “high risk”. Any discrepancies between the reviewers were resolved by a consensus.

The level of evidence was independently evaluated by two reviewers using the SIGN scale, a tool that assesses the quality of studies, assigns evidence levels based on their design and the risk of bias, and classifies them into categories ranging from high-quality systematic reviews (1++) to expert opinions (4) [22]. Any possible discrepancies between the reviewers were resolved by a consensus.

The possibility of retracted trials being inadvertently included in the meta-analysis was verified using the Retraction Watch database of retracted articles, and none of the selected studies raised serious integrity concerns.

### 2.6. Data Extraction

Data extraction was independently carried out by two reviewers. The data sought included authorship, publication year, and territory; type of study; SIGN scale level of evidence [22]; sample size and comparison groups; as well as the objectives, maximum and minimum values, mean, and standard deviation of the intervention results.

The primary outcomes of this study related to glycemic control were glycated hemoglobin (Hb A1c), time in range (TIR), time above range (TAR), time below range (TBR), and coefficient of variation (CV); and those related to emotional well-being were symptoms of stress or anxiety symptoms, quality of life, frequency of family conflicts, social support, eating behavior, and treatment satisfaction. The secondary outcome related to glycemic control was capillary glucose, and those related to emotional well-being were diabetes knowledge, attitudes, self-efficacy, self-care, and adherence to treatment.

### 2.7. Synthesis

The authors performed an analysis using the Review Manager (RevMan) version 5.4 software. Glycemic control was assessed using Hb A1c, capillary glucose, TIR, TAR, TBR, and CV. Emotional well-being was assessed using stress, depression, anxiety symptoms, quality of life, frequency of family conflicts, social support, feeding disorders, and treatment satisfaction.

For some the outcomes mentioned, heterogeneity (I^2^), standardized mean differences (SMDs), and 95% confidence intervals were calculated [23]. Changes in scores from baseline were used for the meta-analysis. The random effects model was used for Hb A1c at 3 and 6 months, TIR, and TAR, while the fixed effects model was considered for Hb A1c at 12 months and for CV. An analysis of the certainty of the evidence, forests plots, and a summary of the findings table were also performed (Supplementary S2) [28].

## 3. Results

### 3.1. Results of the Screening Process

**Figure 1:**
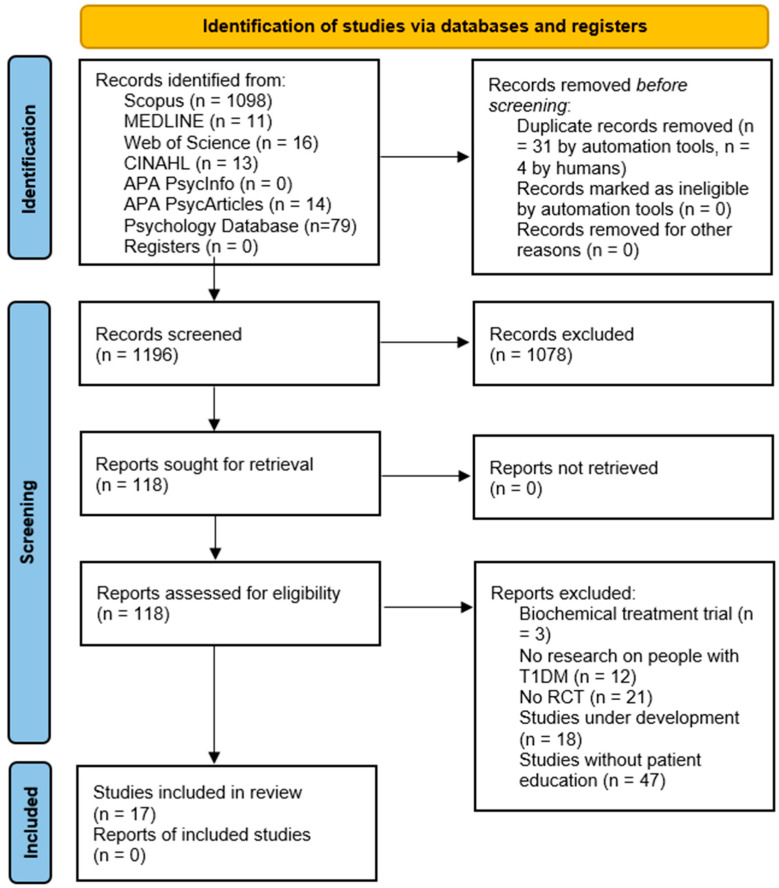
PRISMA 2020 flow diagram. Note: Authors’ own elaboration with PRISMA 2020.

The screening process included removing records automatically (n = 31) and manually (n = 4), excluding references during the title and abstract screening (n = 1078), and discarding them after the full text reading (n = 101) (Figure 1).

### 3.2. Description of the Included Studies

All the included studies (n = 17, 100%) met the eligibility criteria and were RCTs. They were published in Spain (n = 1, 5.88%), the United States (US) (n = 2, 11.76%), Canada (n = 1, 5.88%), the United Kingdom (UK) (n = 1, 5.88%), Iran (n = 3, 17.64%), South Korea (n = 2, 11.76%), Sweden (n = 1, 5.88%), Turkey (n = 1, 5.88%), Germany (n = 1, 5.88%), Poland (n = 2, 11.76%), Brazil (n = 1, 5.88%), and Thailand (n = 1, 5.88%); in 2022 (n = 4, 23.52%), 2021 (n = 4, 23.52%), 2020 (n = 2, 11.76%), 2019 (n = 4, 23.52%), and 2018 (n = 3, 17.64%).

The trials with the largest sample size were Fisher et al. (2018) and Hessler et al. (2021) (n = 301), while the smallest sample size was found in Lee et al. (2022) (n = 34) [29,30,31]. Fisher et al. (2018) and Hessler et al. (2021) had the largest overall sample size (n = 152), and Lee et al. (2022) had the smallest sample size (n = 17), in each investigation arm [29,30,31]. Most of the studies (n = 11, 64.70%) mainly included women in their samples and some (n = 3, 17.64%) had men as their focus.

### 3.3. Risk of Bias in the Included Studies

**Figure 2:**
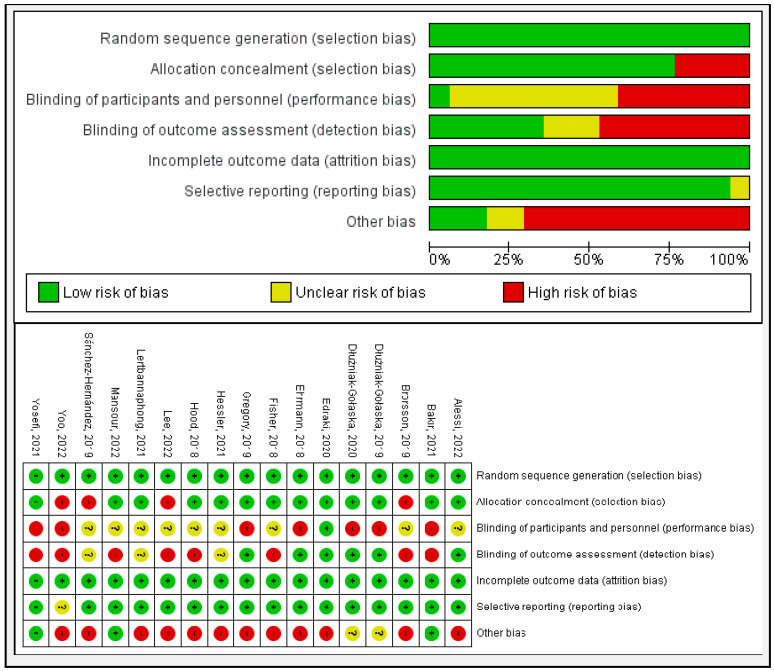
Risk of bias graph and summary. Note: Authors’ own elaboration with RevMan [29,30,31,32,33,34,35,36,37,38,39,40,41,42,43,44,45].

All the RCTs (n = 17) included a low risk of not having a random sequence generation and complete outcome data (n = 17, 100%); most of them (n = 13, 76.47%) had a low risk of not having adequate allocation concealment, and of considering their reporting as selective (n = 16, 94.11%). However, some (n = 8, 47.05%) had a high risk of blinding the outcome assessment or the blinding of participants or personnel (n = 7, 41.17%). The latter was unclear in many trials (n = 9, 52.94%). The section was used to report possible biases arising from financial support in RCTs, which was found in most of the trials (n = 12, 70.58%). This analysis resulted in the assignment of a level of evidence of 2+ to the included studies, according to the SIGN scale [22] in most of them (n = 13, 74.47%) (Figure 2).

### 3.4. Characteristics of the Sample Studied

Most of the studies featured a sample that was predominantly composed of women (n = 11, 64.70%). Some (n = 3, 17.64%) research teams had a sample mainly consisting of men, as observed by Yoo et al. (2022), Gregory et al. (2022), and Dłużniak-Gołaska et al. (2019) [37,38,39]. No studies (n = 17, 100%) differentiated between gender and sex in their results. Additionally, many investigations clarified that their sample included individuals over 18 years old (n = 8, 47.05%), a similar ratio to those who were underage or adolescents (n = 7, 41.17%). The minimum age of the people enrolled in the studies was 9.8 in Gregory et al. (2022), and the maximum age was 45.1 years in Fisher et al. (2018) and Hessler et al. (2021), with a mean of 29.22 years [29,30,38]. The rest of the RCTs (n = 2, 11.76%) did not clarify the age of their sample.

Some trials (n = 13, 76.47%) reported a disease duration with a mean of 10.64, a minimum of 3.41 in Yosefi et al. (2021), and a maximum of 25 years in Lertbannaphong et al. (2021) [32,40]. Furthermore, Bakir et al. (2021) showed that the participants in their sample had no previous diagnosis of T1DM in their families. Together with Ehrmann et al. (2019), they mentioned that the participants in their sample had attended some TPE sessions before the trial [33,41]. Furthermore, certain studies (n = 11, 64.70%) registered the base Hb A1c of the participants in their sample, ranging from a minimum of 8.23% in Dłużniak-Gołaska et al. (2020), to a maximum of 10.3% in Lertbannaphong et al. (2021) and a mean of 9.13% [32,34]. Ehrmann et al. (2018), Dłużniak-Gołaska et al. (2019), and Dłużniak-Gołaska et al. (2020) also registered the baseline body mass index (BMI) of their participants, which was overweight in both groups of the 1st study, 77.5% were underweight in the 2nd study, and 77.2% were between a healthy weight and underweight in the 3rd study [33,34,39]. Some trials (n = 7, 41.17%) explicitly addressed the possible differences between the samples allocated to the control and intervention groups, and all reported no significant differences (*p* < 0.05).

### 3.5. Characteristics of the Different Types of TPE

The TPE sessions were on different topics, like carbohydrate (CH) counting or exchange (n = 12 interventions), insulin dose adjustment (n = 7), the management of technologies (n = 4), problem-solving skills (n = 4), T1DM self-care instruction (n = 9), physical exercise coaching (n = 2), the improvement of emotional well-being (n = 7), and question-solving interviews (n = 7).

These sessions were led by nurses (n = 4, 23.52%); featured audiovisual media (n = 10, 58.82%) such as PowerPoints, food models, crosswords, DVDs, apps, and flyers; were carried out in person, at a distance, or with mixed methodology; were conducted in groups (n = 9, 52.94%) with a maximum of 10 people, as shown by Lertbannaphong et al. (2021), and a minimum of 3 participants (n = 2, 11.76%), as indicated by Ehrmann et al. (2018) and Dłużniak-Gołaska et al. (2020) [32,33,34]. These lasted a minimum of 10 min, as demonstrated by Alessi et al. (2022), and a maximum of 120 min, as represented by Hood et al. (2018) [35,36].

The distribution of the trials was similar in hospitals (n = 5, 29.41%) and health centers (n = 5, 20.41%), followed by clinics (n = 3, 17.64%), and a diabetes association (n = 1, 5.8%). Among the clinics, the majority (n = 2, 11.76%) were affiliated with hospitals; and among the health centers, some (n = 2, 11.76%) were medical centers, while others (n = 3, 17.64%) were specific for diabetes.

### 3.6. Outcomes

#### 3.6.1. Influence of TPE on the Glycemic Control of People with T1DM

The glycemic control was evaluated using Hb A1c in most studies (n = 12, 70.58%). Thus, Fisher et al. (2018) evidenced a statistically significant reduction at 3 months (*p* = 0.01) in the KnowIt and OnTrack groups, without any difference among these investigation arms; Lee et al. (2022) also demonstrated this, as the Hb A1c was lowered from 9.2% to 7.8% (*p* = 0.003) in the intervention group and from 8.6% to 7.8% (*p* = 0.004) in the control group, and Yoo et al. (2022) reported a decrease in the outcome, even though it occurred in both groups (*p* = 0.001 in the control group and *p* < 0.001 in the intervention group) [29,31,37]. Furthermore, Bakir et al. (2021) seconded this affirmation (*p* < 0.001), although only in the Information–Motivation–Behavioral (IMB) Skills group, which is a similar result to that of group E of Dłużniak-Gołaska et al. (2019, *p* = 0.038), who also showed a significant difference between their groups; to that of Mansour et al. (2023), who observed a significant decrease in the outcome (*p* < 0.001) in the intervention group; and to that of Edraki et al. (2020), who also evidenced this (*p* < 0.005) [39,41,42,43]. In fact, Gregory et al. (2019) presented that TPE is significantly effective in reducing Hb A1c when carried out at home or in the hospital (at 63.7 mmol/mol at home and at 6.0 mmol/mol in the hospital, *p* < 0.001) [38]. On the other hand, Lertbannaphong et al. (2021) did not show this significant change at this time in any of the groups (*p* = 0.095) [32].

**Figure 3:**
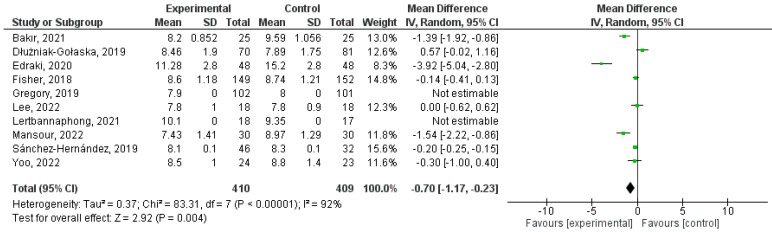
3-month Hb A1c forest plot. Note: Authors’ own elaboration with RevMan [29,31,32,37,38,39,41,42,43,45].

Furthermore, in the statistical analysis, the experimental (TPE) and control (standard care) groups were established, Hb A1c measured at 3 months was determined to be a continuous quantitative variable and a confidence interval (CI) of 95% (−1.77, −0.23), a random effects model and heterogeneity (I^2^ = 92%) between included studies were identified. Thus, it was established that the combined effect of these was statistically significant (*p* = 0.004) (Figure 3).

Hb A1c was also measured at 6 months in some studies. Both Bakir et al. (2021) and Ehrmann et al. (2018) confirmed a significant reduction (*p* < 0.001 in both) in their interventions during this period, with a significant difference (*p* = 0.0041) between the groups of the latter [33,41]. However, both in Brorsson et al. (2019) and in Lertbannaphong et al. (2021), a non-significant improvement of this outcome was shown (*p* = 0.19 and *p* = 0.333, respectively) [32,44].

**Figure 4:**
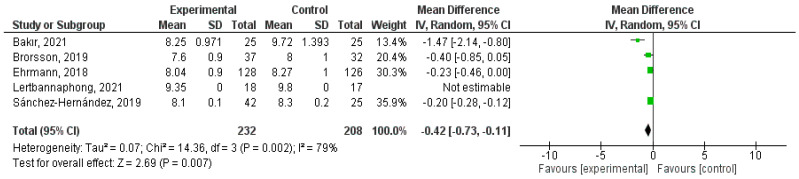
6-month Hb A1c forest plot. Note: Authors’ own elaboration with RevMan [32,33,41,44,45].

The statistic used to determine the effect of TPE on Hb A1c at 6 months was the same as that performed at 3 months, previously described, although with less heterogeneity (I^2^ = 79%) and a different CI, although it was 95% (−0.73, −0.11). Therefore, the significant influence of education on Hb A1c was determined at 6 months (*p* = 0.007) (Figure 4).

Regarding the results of this Hb A1c after 12 months of the educational intervention, Brorsson et al. (2019) showed a significant difference between the Guided Self-Determination (GSD-Y) group and standard care (SC) (7.8% GSD-Y, 8.6% SC, *p* = 0.009). On the other hand, Sánchez-Hernández et al. (2022) stated that the Hb A1c levels were not significantly different between the intervention and control groups (64 ± 1.3 mmol/mol (8.0 ± 0.1%) in the intervention group, 68 ± 1.6 mmol/mol (8.4 ± 0.1%) in the control group, *p* = 0.081) [44,45]. Similarly, Gregory et al. (2019) did not report a significant difference at 24 months in the at-home or hospital TPE (72.1 mmol/mol and 72.6 mmol/mol, respectively, *p* = 0.863) [38].

**Figure 5:**
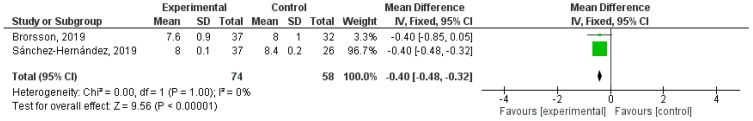
12-month Hb A1c forest plot. Note. Authors’ own elaboration with RevMan [44,45].

Unlike the previous analysis, the Hb A1c at 12 months had a different 95% CI (−0.48, −0.32), null heterogeneity between the studies (I^2^ = 0%) was found, and the fixed effects model was used. The other statistical characteristics were identical to those of the previous comparisons. Therefore, a significant effect of the intervention was established (*p* < 0.001) (Figure 5).

Glycemic control was also assessed using capillary blood glucose and continuous glucose monitoring (CGM) sensor values in various studies (n = 4, 23.52%). Hood et al. (2018) showed that the capillary blood glucose did not experience a significant difference (*p* > 0.05) between the Penn Resilience Program (PRP T1D) and the Advanced Diabetes Education group (9.2 ± 2.0, and 9.0 ± 1.8, respectively) at 12 months, although Mansour et al. (2022) reported a significant reduction in fasting and postprandial blood glucose at 3 months (*p* < 0.001) [36,42].

Similarly, Lee et al. (2022) reported that TIR improved significantly at 3 months in the intervention group (*p* = 0.001) and that TBR, TAR, and CV also decreased with statistical significance (*p* = 0.002, *p* = 0.026, and *p* = 0.018, respectively) [31]. Likewise, Yoo et al. (2022) proposed that there were no significant differences between the groups in TBR (*p* > 0.05) and that there was no significant improvement in TIR or CV in the intervention group [37]. However, they showed that TAR was significantly higher in the control group than in the intervention (*p* < 0.001), at the same time.

**Figure 6:**
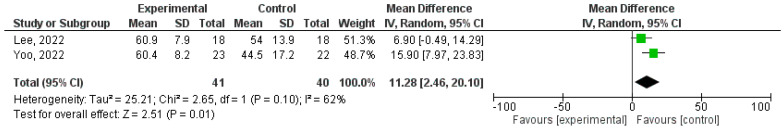
3-month TIR forest plot. Note: Authors’ own elaboration with RevMan [31,37].

Regarding the statistical analysis of the influence of TPE on the variables of the ambulatory glucose profile (AGP) report at 3 months, the experimental and control (standard care) groups were established: (1) TIR, (2) TAR, and (3) CV values were represented as continuous quantitative variables, and a 95% CI was selected. In the first outcome, a 95% CI (2.46, 20.10) was determined, the random effects model was used, and a high heterogeneity was established between the compared studies. The combined effect of these RCTs on the effect of TPE was statistically significant in the control group (*p* = 0.01) (Figure 6). In the second variable, TAR (blood glucose >181 mg/dL), the same statistics were performed as in TIR, although a greater heterogeneity was observed (I^2^ = 77%) and a different 95% CI (−26.64; 1.18) was calculated. However, on this occasion, no statistical significance could be found in the intervention group (*p* = 0.07). In the statistical analysis referring to the third result, CV, a CI of 95% (−6.62, 0.57) was established, a fixed effects model was used, and there was no heterogeneity between the RCTs (I^2^ = 0%). No significant differences were found at 3 months (*p* = 0.10) in the intervention group.

Associations between the alteration of different variables with changes in Hb A1c were also highlighted, such as those reported by Fisher et al. (2018) on the statistical significance between stress reduction and Hb A1c at 9 months (*p* = 0.01), although without a difference between groups; or the reduction of Hb A1c, associated with the reduction of family conflicts related to pathology in the intervention group (*p* = 0.019) [29]. Furthermore, Lee et al. (2022) highlighted that the intervention group showed a correlation between glycosylated changes with some sensor measurements, such as TIR (*p* = 0.006) or TAR (*p* = 0.001), 3 months after education [31].

#### 3.6.2. Effects of Structured TPE on Emotional Well-Being in People with T1DM

Emotional well-being was studied using various variables in RCTs (n = 9; 52.94%), such as (1) the level of stress or overload caused by diabetes, (2) depressive or anxious symptoms, (3) quality of life, (4) frequency of family conflicts, (5) social support, (6) alterations in eating behavior, and (7) treatment satisfaction.

Regarding the first of these, Fisher et al. (2018) showed a significant reduction in their groups at 3 and 9 months (*p* < 0.02), measured using the Type 1 Diabetes Distress Scale (T1-DDS), as well as a decrease in stress due to hypoglycemia (*p* < 0.03) [29]. Hessler et al. (2021) also showed a decrease in stress on a subscale of the T1-DDS (*p* < 0.05), which even established that there is no direct relationship between stress reduction and glycemic control after 9 months from the educational intervention [30]. However, Hood et al. (2018) did not achieve statistical significance in any of their groups (*p* > 0.05) with this variable, measured using the Problem Areas in Diabetes-Teen (PAID-T) at 12 months, a similar result to that obtained by Alessi et al. (2022) after 4 months, using the Diabetes Mellitus B-PAID Questionnaire (Diabetes B-PAID, *p* > 0.05); and that of Brorsson et al. (2019), which showed that there were no significant differences at 6 and 12 months in overload for any of their groups (*p* > 0.05), according to the Check your Health questionnaire [35,36,44].

Regarding the second outcome, a significant decrease was observed in both groups of Lee et al. (2022) with no differences between them, assessed according to the Patient Health Questionnaire-9 (PHQ-9), after 3 months [31]. In fact, Hood et al. (2018) found no significant differences in depressive symptoms between the two groups in their study (8.5 ± 8.7 and 7.3 ± 6.9, *p* > 0.05), according to the Children’s Depression Inventory, at 12 months [36]. Regarding anxiety symptoms, Lee et al. (2022) reported a significant decrease in this in the intervention group at 3 months, measured using the General Anxiety Disorder-7 (GAD-7) questionnaire [31].

Quality of life, the third of the variables studied, was assessed by Brorsson et al. (2019), who did not find any significant differences at 6 and 12 months between the intervention and control groups, as measured using the DIABKIDS (*p* > 0.05) and corroborated by Dłużniak-Gołaska et al. (2020), according to the PedsQL scale. However, in the latter study, a significant reduction in the communication subscale was verified in the control group (*p* = 0.038) and a significant difference between the groups was observed (*p* = 0.007), as well as a marked improvement in the same subscale in the intervention group, compared with group C (*p* = 0.0014), after 6 months.

Furthermore, Brorsson et al. (2019), with respect to the fourth outcome, referring to the number of family conflicts, showed that there were no significant differences between their groups at 6 and 12 months, measured using the Diabetes Family Conflict Scale (DFCS) (*p* > 0.05) [44].

The same research team, regarding the fifth outcome, related to social support, determined that there were no significant differences between the groups, according to the Multidimensional Scale of Perceived Social Support (MSPSS) (*p* > 0.05) at 6 months after the intervention.

On the other hand, in the sixth outcome, Alessi et al. (2022) showed that, after having measured attitudes related to eating using the Eating Attitudes Test (EAT-26) at 16 weeks from the start of the TPE, that the scores in the control group increased from 72.4% to 75.9%, while in the intervention they were reduced from 72.4% to 62.1%, although without statistical significance (*p* = 0.26) [35].

Regarding the seventh variable on treatment satisfaction, Yoo et al. (2022) observed a significant difference in the intervention group at 3 months using the Diabetes Treatment Satisfaction Questionnaire (DSTQ) (*p* < 0.001); which agreed with Sánchez-Hernández et al. (2019), who evaluated using the Spanish version of the DTSQ at 3, 6, and 12 months in their ANAIS group (*p* = 0.0001) [37,45]. However, Lee et al. (2022) showed this on the satisfaction subscale of the Korean version of the DTSQ (*p* = 0.016), although this improvement was not such, compared with the rest of the subscales of the questionnaire, after 3 months [31].

#### 3.6.3. Influence of TPE on Knowledge, Attitudes, Self-Efficacy, Self-Care, and Adherence to Treatment in People with T1DM

The level of knowledge about different aspects of the pathology, the first of the variables, was measured by certain publications (n = 3; 17.64%). Of these, Bakir et al. (2021) showed that this variable was significantly higher in their intervention group at 6 months, after having measured it using the Diabetes Information Evaluation Form (DIEF, *p* < 0.001) [41]. This was corroborated by Dłużniak-Gołaska et al. (2019), who reported a significant improvement (*p* = 0.042) in both groups in the same period, in the sections ‘healthy eating’, ‘CH count’, ‘glycemic response to food’, and ‘reading labels’ from the Nutrition Knowledge Survey (NKS); and by Lertbannaphong et al. (2021), who showed that, in the same span, the knowledge of T1DM improved with statistical significance in their DSME and DSME+IM groups (from 19 to 21 and from 18.5 to 21, respectively; *p* = 0.001), according to the Siriraj Diabetes Knowledge Test [32,39].

The second of the variables mentioned, self-esteem, also demonstrated a statistically significant increase in the study by Bakir et al. (2021) at 6 months, who evaluated this variable using the Child Attitude Toward Illness Scale (CATIS, *p* = 0.001) [41].

Furthermore, regarding the third variable studied by several RCTs (n = 3, 17.64%), Brorsson et al. (2019) established that there was no significant difference between their groups after having assessed self-efficacy at 6 and 12 months, although Yosefi et al. (2021) showed a significant increase in this variable in both groups after the first session and 1 month after it, having measured it using the Diabetes Management Self-Efficacy Scale (DMSES) (27.97 ± 5.08, 41.46 ± 4.41, and 44.55 ± 4.38 in the intervention group; and 28.864 ± 4.83, 29.1 ± 4.9, and 29.37 ± 4.61 in the control group) [40,44]. Similarly, Bakir et al. (2021) showed a significant increase in self-efficacy in their intervention group according to the DMSES (*p* = 0.001), at 6 months [41].

The self-care behaviors, represented as the fourth outcome, were reviewed by Hood et al. (2018), who showed, through the Self-Care Inventory, that their intervention group had a mean higher than that of their control group at 12 months, although without significant differences (57.2 ± 9.3 and 52.4 ± 9.2, respectively) [36]. However, Hessler et al. (2021) achieved a statistically significant association between six diabetes self-care subscales between self-care and stress level reduction (*p* < 0.05), which was greater in OnTrack than in KnowIt, at 9 months of the study [30]. This significance is related to the observation by Edraki et al. (2020), whose intervention group reported a significant improvement in self-care behaviors according to the Self-Care Questionnaire (*p* < 0.001), after 3 months [43].

In the fifth variable investigated, the adherence to treatment in people with T1DM, Mansour et al. (2022) showed a significant increase (*p* < 0.001) on average at 3 months in the intervention group, according to the Modanloo adherence to treatment questionnaire (MATQ) [42].

## 4. Discussion

The positive effect of structured TPE has been established on different glycemic control variables, such as Hb A1c, fasting or postprandial capillary blood glucose, and the values collected by the CGM sensors, such as TIR, TAR, TBR, and CV. This influence has been observed with statistical significance in some studies at 3, 6, and 12 months in the case of the first variable (e.g., Hb A1c reduction at 3 months: 95% CI [−1.77, −0.23], *p* = 0.004), and at 3 months in the second and last three variables. In fact, in the meta-analysis of the first outcome, a significant difference has also been shown at 3, 6, and 12 months (e.g., Hb A1c reduction at 6 months: 95% CI [−0.73, −0.11], *p* = 0.007; Hb A1c reduction at 12 months: 95% CI [−0.48, −0.32], *p* < 0.001). However, among the AGP variables, only the statistical analysis of the TIR has shown this significance at 3 months (TIR: 95% CI [2.46, 20.10], *p* = 0.01). Significant associations have also been verified between these outcomes and others of a psychosocial nature, such as stress and Hb A1c levels at 9 months, Hb A1c with family conflicts, and hemoglobin with TIR and TAR, 3 months after intervention.

This type of intervention also has some influence on emotional variables, such as stress, depressive or anxious symptoms, quality of life, frequency of family conflicts, social support, eating behavior disorders, and treatment satisfaction. Most authors have not significantly verified in the first, as this has also occurred with depressive symptoms in the second at 3 and 12 months, in the third at 6 and 12 months, and in the fourth or in the fifth. However, a significant decrease in anxiety has been reported at 12 weeks, or in different anxiety assessment subscales (*p* < 0.05). Evidence of the effects of TPE on knowledge, self-esteem, self-efficacy, self-care, and adherence to treatment has been provided. Therefore, there is a consensus on statistical significance with respect to the first, second, and fifth variables. Some of the trials corroborated this condition for the third and fourth outcomes (e.g., self-care improvement: *p* < 0.05) [30,36,42,43].

Another review published in 2023 evaluated the effectiveness of a standardized nursing process [46] using NANDA-I, NIC, and NOC taxonomies [9,10,11]. It included 17 studies, in which: (a) the most frequently used NANDA-I diagnoses were Risk for Infection, Acute Pain, Self-Care Deficit, Impaired Physical Mobility, and Anxiety; (b) the most implemented NIC interventions were Pain Management, Infection Prevention, Assistance with Self-Care, Promotion of Mobility, and Anxiety Reduction; and (c) the most commonly assessed NOC outcomes were Pain Control, Infection Status, Level of Independence in Self-Care, Mobility Capability, and Anxiety Level. The results showed significant improvements in diagnostic decision-making and health outcomes (*p* < 0.05 in multiple studies), highlighting the impact of these standardized nursing taxonomies. However, there was no evaluation of the effectiveness of the intervention or patient satisfaction, and there was a scarcity of diabetes-related diagnoses, objectives, and interventions, indicating the need for further research [46].

Due to this, the present systematic review and meta-analysis stands out by focusing exclusively on T1DM, addressing a critical gap in the literature left by previous systematic reviews that combined the results from different types of diabetes without distinguishing between them. In doing so, it highlights the unique challenges and benefits associated with T1DM-specific interventions. Furthermore, the results obtained in this study underscore the importance of health promotion in managing a chronic disease such as diabetes mellitus and have great relevance in their contributions to clinical care, research, and nursing practice. The role of structured medical education in glycemic control and quality of life is particularly evident, because by equipping patients with essential self-management skills and fostering adherence to treatment, structured TPE helps to bridge care gaps, improving both the clinical outcomes and psychosocial dimensions of living with T1DM.

This dual impact underscores the need to integrate structured TPE into routine care for people with T1DM. Thus, the development of a research line around structured TPE led by nurses becomes notable to improve the biopsychosocial well-being of people with T1DM. Therefore, the development of a larger number of RCTs is suggested, to corroborate the optimal characteristics that education must have to achieve statistical significance in all the specific variables of pathology, and the sociodemographic characteristics of the sample in which it produces a greater effect; to favor the use of new T1DM technologies, designed using biomedical engineering; and to develop a structured effective structured TPE in the field of primary care, which is absent in the current literature.

### Strengths and Limitations

This study has several limitations, such as heterogeneity among studies, which can limit the generalizability and interpretation of the meta-analysis. It is important to note that most of the included studies were from developed countries, which can introduce a geographic bias and limit the applicability of the findings in developing regions. The need to exclude many trials due to errors in their randomization or the lack of differentiation of their results based on diabetes type or other characteristics related to glycemic control and emotional well-being is noteworthy. Furthermore, including a sample without an age limitation in this study may introduce confounding variables that affect both glycemic control and emotional well-being; however, this was necessary due to the small number of RCTs found (n = 10) after a title and abstract screening during a pilot search analyzing the effect of TPE in adolescents or pediatric people with T1DM. It should be noted that some of the included trials did not adequately blind the participants or personnel, which could introduce performance bias and affect the reliability of the results. The need to include trials that determine the impact of the disease on eating behavior is also appreciated, to the detriment of a body image analysis, given the small number of RCTs that study this outcome. The use of the term ‘health education’ instead of TPE in the search strategy, due to the lack of a specific entry for the mentioned in MeSH, is also worth mentioning; however, this was not a drawback, as all the selected trials were about TPE. Finally, the small number of RCTs that provided an analysis of the results carried out with the same measurement system is highlighted, as this influenced the variables available for study in the meta-analysis.

Despite these limitations, some strengths stand out, such as that this study synthesizes RCTs, which is the gold standard in clinical research and therefore enhances the validity and reliability of its findings. It is relevant to highlight the exhaustive record of the reason for the exclusion of all the documents analyzed from the title and abstract screening stage, which is a differential factor in relation to other similar documents. Therefore, the development of a research protocol and its registration in PROSPERO [20] are also established as a guarantee of methodological quality, as well as the use of four official verifications to avoid any possible bias in the study, and the complete registration of the documents used in the screening process and in the various PRISMA and AMSTAR 2 checklists [23,25], which ensures the transparency and internal consistency of the research. It is also stated that the results obtained are of high quality, due to all the studies included being RCTs, most of them having a level of evidence of 2+ on the SIGN scale [22], and the absence of a conflict of interest of the author, or any type of research funding.

## 5. Conclusions

This systematic review shows that structured TPE has a significant positive impact on glycemic control and emotional well-being in people with T1DM. The findings highlight improvements in key areas such as HbA1c levels, anxiety, and stress management, as well as advances in knowledge, self-efficacy, and treatment adherence.

However, the heterogeneity between studies and the lack of data on certain results limit the generalizability of the findings. Despite these limitations, this review emphasizes the importance of nurse-led TPE programs tailored to the specific needs of people with T1DM, incorporating structured methodologies that address both the clinical and psychosocial dimensions of care.

This review strengthens the role of TPE as an essential tool in the comprehensive treatment of chronic conditions like T1DM, while also highlighting the need for more randomized controlled trials to refine its implementation and maximize its clinical impact.

## Figures and Tables

**Table 1:** Search strategy and databases.

Search Strategy	Databases
(“Type 1 Diabetes Mellitus” OR Diabetes) AND (“Health Education” OR “Health Promotion” OR “Patient* Education As Topic” OR “Educat*”) AND (“Glycemic Control*” OR “emotional well-being” OR “Quality of Life” OR “Body Image*” OR “Self-Car*”) AND (“Randomized Controlled Trial”) NOT (“systematic review*” OR “review*” OR “meta-analysis” OR “protocol” OR “COVID*” OR “coronav*” OR “pregnan*”)	Scopus
	MEDLINE
	Web of Science
	CINAHL
	APA PsycInfo
	APA PsycArticles
	Psychology Database

Note: Authors’ own elaboration. The search strategy was the same in all the databases except for Scopus, which was the only one that had some particularities. Due to this, NOT was changed to AND NOT in that database.

**Table 2:** Eligibility criteria.

	Inclusion Criteria	Exclusion Criteria
P	People with T1DM	Pregnant patients
I	RCTs Published in the past 10 years (2014–2024) Available in English or Spanish	Biased randomization
C	N/A	N/A
O	Complete results analysis	Studies in development Questionnaire validation studies Biochemical treatments Results of religious or social events

Note: Authors’ own elaboration. N/A: not applicable.

## Data Availability

No new data were generated in this study. Data sharing is not applicable in this article.

## Supplementary Materials

The following supporting information can be downloaded at: https://www.mdpi.com/article/10.3390/healthcare12232461/s1, Supplementary S1: PRISMA 2020 Checklist; Supplementary S2: Summary of findings.
