# Supplementary material for: Health Promotion in Glycemic Control and Emotional Well-Being of People with Type 1 Diabetes Mellitus: A Systematic Review and Meta-Analysis
Source: Healthcare (Basel). 2024 Dec 6;12(23):2461. doi: 10.3390/healthcare12232461 (PMC11641254; doi:10.3390/healthcare12232461)
Supplement: Supplementary file 1 [file healthcare-12-02461-s001.zip › healthcare-3333995-S2.pdf]

Supplementary S2. Summary of findings.

Authors, publication year and territory	Type of study, SIGN scale and sample size	Outcomes	Follow-up	Educative interventions characteristics	Population and health service or care level
Fisher et al.<br>2018.<br>US.	RCT.<br>SIGN 2+.<br>Total (n=301).<br>OnTrack (n=152).<br>KnowIt (149).	Stress<br>Hb A1c.	3 and 9 months.	KnowIt: advice on causes of T1DM, CH counting, and problem-solving skills.<br>OnTrack: improving emotion regulation skills. 4 online meetings.<br>Both require 1 group workshop with a certified diabetes educator and 1h online meetings for 3 months.	45.1 years, 69.1 % women, 8.80 % (73 mmol) mean Hb A1c at baseline.
Hood et al.<br>2018.<br>US.	RCT.<br>SIGN 2+.<br>Total (n=264).<br>PRP T1D (n=133).<br>*Advanced Diabetes Education* (EI, n=131).	Stress.<br>Depressive symptoms.<br>Self-care.<br>Capillary glycemia.	12 months.	PRP T1D: delivered by diabetes nurses and educators. Prevents depression and reduces stress. Cognitive-behavioral and problem-solving skills. Group format.<br>EI: education about nutrition, exercise, insulin function and diabetes technologies.<br>Both were 9 biweekly sessions of 90-120 min.	Mean age of 15.7 ± 1.1 years, mean T1DM duration of 6.9 ± 4.0 years, baseline Hb A1c of 9.1 ± 1.9 %, 60 % female and 1/3 belonging to ethnic minorities.
Brorsson et al.<br>2019.<br>Sweden.	RCT.<br>SIGN 2-.<br>Total (n=71).<br>GSD-Y (n=37).<br>*Standard Care* (SC) (n=34)	Hb A1c.<br>Quality of life.<br>Stress.<br>Family conflicts.<br>Self-efficacy.	6 and 12 months.	GDS-Y: problem-solving skills. Group format. 7 sessions of 2 hours.<br>Both groups received introductory training on the insulin pump.	Women (59.4 %) of 12.9 years and 5.0 years of disease duration, baseline Hb A1c of 8.6 %.<br>Hospital.
Bakır et al.<br>2021.<br>Turkey.	RCT.<br>SIGN 2+.<br>Total (n=50).<br>IMB (n=25).<br>Control (n=25).	Social Support.<br>Hb A1c.<br>Knowledge.<br>Attitudes.<br>Self-efficacy.	Baseline and 6 months.	IMB: improve diabetes knowledge, motivation, behavioral skills, and self-efficacy. 8 home visits.	There were no significant population differences between the groups. People around 14 years old without a previous diagnosis of diabetes in the family, who have attended more than 3 diabetes education sessions previously, and a duration of the disease of more than 5 years.<br>Hospital clinics.

Hessler et al. 2021. Canada.	RCT. SIGN 2+. Total (n=301). OnTrack (n=152). KnowIt (n=149).	Stress. Self-care. Glucose monitoring.	9 months.	KnowIt: led by a diabetes educator. Education on the causes and management of T1DM. OnTrack: led by a psychologist with experience in T1DM. Education about the emotional side of DM.	69.1 % women, 45.1 years on average, initial Hb A1c of 8.8 %, 24.5 years on average with T1DM.
Mansour et al. 2022. Iran.	RCT. SIGN 2+. Total (n=60). Family education (n=30). Routine Care (n=30).	Fasting blood glucose. Hb A1c. Adherence to treatment.	3 months.	Intervention group: identification of the strengths and weaknesses of patients and relatives. 12 sessions of 90 min Both groups received 1 introduction and 3 routine education sessions per week.	Individuals aged 21.22 years, 60.7 kg, and 9.42 years of disease duration. There were no significant differences between the subjects of both groups. Diabetes association.
Ehrmann et al. 2018. Germany.	RCT. SIGN 2+. Total (n=268). *Insulin Pump Treatment* (INPUT) (n=135). Control (n=133).	Hb A1c. Incidence of severe hypoglycemia.	6 months.	Intervention: SC, clinical, technological, and psychosocial components, basal profiles programming, prandial insulin adjustment, recognition of problematic patterns in their blood glucose, usage of the insulin pump, resolution of emotional obstacles. 12 sessions of 90 min. Group format of 3 to 8 participants per group.	Men (44 in INPUT and 64 in control) and women (91 in INPUT and 69 in control), 45 years old in both groups, 11.4 years of education, a mean duration of diabetes of 21 years of 28, BMI 2 in INPUT and 27.9 in control, and an initial Hb A1c of 8.3 % in both groups. Centers specialized in secondary care on CSII.
Edraki et al. 2020. Iran.	RCT. SIGN 2+. Total (n=96). Intervention (n=48). Control (n=48).	Hb A1c. Self-care.	3 months.	Intervention: 4 sessions of 90 min for 4 weeks. Group format with 6 subgroups of 8 people. Face-to-face training, questions and answers, group discussions, role-play, and scientific books.	61.90 % of women with a mean age of 14.85 in the intervention group and 15.02 in the control group, 91.70 % of people had no T1DM side effects, disease duration of 5.26 years in intervention and 5.24 in control. There was no significant difference between the 2 groups. Center for diabetic patients.
Dłużniak-Gołaska et al. 2019. Poland.	RCT. SIGN 2+. Total (n=196). Experimental (E, n=98).	Hb A1c. Knowledge.	3 and 6 months.	Control intervention: informational class. Experimental group: informational class, true-false questionnaires and apps about balanced diet, food label, product photos	55.6 % of men with 95 % GC using a glucometer, 86.8 % perform calculation of CH exchanges, 66.9 % report several episodes of hypoglycemia per month and 79.5 % several episodes of

	Control (C, n=98).			for the calculation of HC exchange. 60 min.	hyperglycemia per day, 99.3 % do not suffer from ketoacidosis, 77.5 % with low BMI.<br>There were no significant differences between the groups in most of the variables.<br>Hospital.
Dłużniak-Gołaska et al.<br>2020.<br>Poland.	RCT.<br>SIGN 2+.<br>Total (n=170).<br>Experimental (E, n=85).<br>Control (C, n=85).	Quality of life.<br>Communication.<br>Physical activity.<br>diabetes symptoms.<br>Knowledge.	6 months.	Control: traditional 30-min class. Theoretical knowledge of nutrition<br>Experimental: nutrition education, true-false questionnaire, various photographs of CH exchanges, VitaScale answer verification. Groups of 3-5 people. 90 min. Led by a dietitian educator.	58.8 % of women with 51.5 % of moderate physical activity and underweight or normal in 77.2 % BMI, 13.72 years old, Hb A1c of 8.23, disease duration of 5.58 years, PedsQL of 64.04, ATT of 71.56, worry of 59.44, and diabetes symptoms of 59.86.<br>Hospital.
Alessi et al.<br>2022.<br>Brazil.	RCT.<br>SIGN 2+.<br>Total (n=58).<br>Intervention (n=29).<br>Control (n=29).	Emotional disorders.<br>Stress.<br>Sleep quality.<br>Eating disorders.	16 weeks.	Intervention: telephone educational interventions. 10 min. GC, diet, emotional overload, healthy habits, and ATT.<br>Control: standard care and a website with recommendations about healthy habits.	55.2 % of women, 43.8 years old, disease duration of 25 years, with an average Hb A1c of 8.7 %, with the presence of diabetic retinopathy. There were no significant differences between the groups.<br>2 public centers.
Yosefi et al.<br>2021.<br>Iran.	RCT.<br>SIGN 2+.<br>Total (n=70).<br>Intervention (n=35).<br>Control (n=35).	Self-efficacy.	1 month.	Intervention: 8 sessions of 60 min for 4 weeks. Face-to-face and in groups format. Educational objectives, conditions, discussions, and questions. PowerPoints or videos.<br>Both groups received online care for T1DM.	58.2 % of women aged 14.99 years, disease duration of 3.41 years, and a daily insulin intake of 53.5 U on average. There was no significant difference in the sociodemographic characteristics of each group.<br>diabetes center.
Lee et al.<br>2022.<br>South Korea.	RCT.<br>SIGN 2-.<br>Total (n=34).<br>Intervention (n=17).<br>Control (n=17).	Hb A1c.<br>TIR.<br>TAR.<br>TBR.<br>CV.<br>Treatment satisfaction.	3 months.	Intervention: 10-min sessions. Conversion of HC to insulin dose, HC count, insulin management, identifying the causes of hyperglycemia, hypoglycemia or glycemic variability, lifestyle advice and usage of the CGM sensor.	52.8 % of the study subjects were women, with an age of 44.3 years and a mean duration of disease of 17.1 years. No significant differences were observed between the recruited groups.<br>Medical Center.

		Depression.<br>Anxiety.			
Yoo et al.<br>2022.<br>South Korea.	RCT.<br>SIGN 2-.<br>Total (n=47).<br>Intervention (n=24).<br>Control (n=23).	Hb A1c.<br>TBR.<br>TAR.<br>TIR.<br>CV.<br>Treatment satisfaction.	3 and 12 months.	Intervention: 3 sessions. In person or by call. Nurse-led. Postprandial insulin dosing, GH counting, and use of CGM systems.	51.1 % were men, 38.6 ± 12.0 years old, disease duration 9.6 ± 6.5 years, and 8.6 ± 1.2 % Hb A1c (70 ± 13.1 mmol/ mole) on average.<br>Medical Center.
Gregory et al.<br>2019.<br>UK.	RCT.<br>SIGN 2+.<br>Total (n=203).<br>Home (n=101).<br>Hospital (n=102).	Hb A1c.<br>Self-esteem.	3 and 24 months.	Intervention: dietetic education and supervision of insulin injection technique. Face-to-face. Nurse-led. Minimum of 3 days, no more than 2 hours. Both groups were given written information.	53.7 % of men aged 9.8 years, with a mean initial Hb A1c of 116.2 mmol/mol.<br>Home visits and hospital.
Sánchez-Hernández et al.<br>2019.<br>Spain.	RCT.<br>SIGN 2-.<br>Total (n=78).<br>ANAIS (n=46).<br>Control (n=32).	Hb A1c.<br>Treatment satisfaction.<br>Freedom of diet.<br>Achievement of personal goals.	3, 6 and 12 months.	ANAIS: self-care, freedom of diet, intensive insulin regimen, CH counting, types of food, and avoidance of hypoglycemia. 5 days and 7 hours. Group format.	55.1 % of women aged 34.6 ± 11.3 years, with 12 years of disease duration and an Hb A1c of 67 ± 5 mmol/mol (8.3 ± 1.0 %) on average.<br>Hospital.
Lertbannaphong et al.<br>2021.<br>Thailand.	RCT.<br>SIGN 2+.<br>Total (n=39).<br>*Diabetes Self-Management Education* (DSME, n=20).<br>DSME with motivational interview (DSME+MI, n=19).	Hb A1c.<br>Dietary control.<br>Self-care.<br>Physical activity.<br>Self-monitoring.<br>Knowledge.	6 months.	DSME: nurse-led. 60-90 min. Groups of 8-10 patients. Self-monitoring of blood glucose, exercise with DM, management of hypo and hyperglycemia, GH count, healthy eating and food exchanges. Food models, questions, and clinical cases.<br>DSME+MI: in addition to the above, they received a motivational interview conducted by endocrinologists and a diabetes education nurse. 2 individual sessions of 45-60 min were held by telephone call at 4 and 5 months, and 1 group session at 3 months.	People who underwent DSME and DSME+MI had an age of 14.18±2.02 and 14.06±2.88 years, with a disease duration of 5.25 and 5.08 years and an Hb A1c of 10.3 % and 9.45 %, respectively.<br>Diabetes clinic attached to a hospital.

Note. Authors' own elaboration.
